# The relationship between individual-level socioeconomic status and preference for medical service in primary health institutions: a cross-sectional study in Jiangsu, China

**DOI:** 10.3389/fpubh.2023.1302523

**Published:** 2024-01-11

**Authors:** Chunxia Miao, Xin Fang, Hong Sun, Yani Yin, Bo Li, Wenxing Shen, Jie Chen, Xiaojing Huang

**Affiliations:** ^1^School of Management, Xuzhou Medical University, Xuzhou, Jiangsu, China; ^2^School of Economics and Management, Nanjing Forestry University, Nanjing, Jiangsu, China; ^3^Personnel Department, The Affiliated Hospital of Xuzhou Medical University, Xuzhou, Jiangsu, China; ^4^Nursing Department, Children's Hospital of Fudan University, Shanghai, China

**Keywords:** primary health institutions, medical service, socioeconomic status, interaction effects, preferences

## Abstract

**Background:**

While China's primary health care (PHC) system covers all citizens, the use of medical services supplied by primary health institutions (PHIs) is not at ideal levels. This study explored the impact of socioeconomic status (SES) on residents' first choice of medical services provided by PHIs.

**Methods:**

This community-based, cross-sectional study was conducted in Jiangsu Province, China, from October 2021 to March 2022. A custom-designed questionnaire was used to evaluate 4,257 adults, of whom 1,417 chose to visit a doctor when they were sick. Logistic regression was used to test the relationships among SES, other variables and the choice of medical services, and interaction effects were explored.

**Results:**

A total of 1,417 subjects were included in this study (48.7% female; mean age 44.41 ± 17.1 years). The results showed that older age (*p* < 0.01), rural residence (*p* < 0.01), a preference for part-time medical experts in PHIs (*p* < 0.01), and lack of coverage by basic medical insurance (*p* < 0.05) were associated with the first choice to use PHIs. In the multiple logistic regression model, SES was not associated with the first choice of medical services supplied by PHIs (*p* > 0.05), but it interacted with three variables from the Commission on Social Determinants of Health Framework (material circumstances, behaviors and biological factors, and psychosocial factors).

**Conclusion:**

Vulnerable individuals who are the target visitors to PHIs are older, live in rural areas, and suffer from chronic diseases. SES, as a single factor, did not impact whether medical services at PHIs were preferred, but it mediated relationships with other factors.

## Introduction

In 2009, China launched a major health care reform and pledged to provide all citizens with equal access to primary health care (PHC) (1). Since then, to support the development of PHC, the Chinese government invested more than 90 billion yuan to construct primary health institutions (PHIs) during 2012–2018 (2). By the end of 2021, nearly 977.8 thousand PHIs of all forms had been established in China, employing more than 4.43 million health workers to cover all communities. According to the sixth health service survey (3), 90% of families in China are within 15 min of a PHI. PHIs are important organizations that provide essential public health services, such as health management and primary medical services for common diseases, to all residents (4). To increase the utilization of medical services supplied by PHIs, China implemented a policy of providing a first diagnosis at PHIs in 2015. However, Chinese individuals decide whether to go to a hospital or a PHI. This means that approval of a transfer from a general practitioner is not necessary for individuals to go to a hospital to seek medical treatment (5). Before the establishment of PHIs, Chinese individuals were accustomed to using medical services in hospitals, and this practice continues to affect the choices of individuals currently (6). To date, the quality of PHC services has not yet been extensively assessed (7). One study showed that of 1,507 participants from three provinces, 55.1% were willing to make their first visit to PHIs (5), and another study confirmed that 2/5 of older patients in China bypassed PHIs to seek care from higher tier facilities (8). Therefore, it is important to study the factors that affect whether individuals seek medical treatment from PHIs.

Residents' use of health services is affected by many factors, which can be classified according to the conceptual framework of Andersen's behavioral model into three categories: predisposing, enabling and need (9). Among these categories, individuals' sociodemographic characteristics are important, and many studies have reported that different populations display heterogeneous sociodemographic characteristics and health care utilization (10). In specific social contexts, individuals are assigned to different social positions, and social stratification in turn engenders differential exposure to unhealthy conditions and differential vulnerability in terms of health conditions and material resource availability (11). Common sociodemographic characteristics that affect the utilization of medical services include age, gender, income, education level, chronic disease diagnosis, perceived health, medical insurance and current place of residence (12–14).

A growing body of literature has demonstrated the complexity of the relationship between socioeconomic status (SES) and the utilization of PHC services. While studies have used varying definitions of SES, most include education, income, and occupation (15–17). SES has an important impact on the utilization of PHC services, with one study reporting that socioeconomic conditions determine whether people utilize PHC services (18). There is a negative correlation between SES and the utilization of PHC services, which means that compared to people with high SES, people with low SES are more inclined to access PHC (19–21).

SES partly explains health care service utilization. The Commission on Social Determinants of Health (CSDH) framework regards material circumstances, behavioral and biological factors, and psychosocial factors as intermediate determinants. This framework assumes that the distribution of these intermediary factors constitutes the primary mechanism through which SES generates health inequities (11). Intermediate determinants dictate differences in the exposure and vulnerability of people based on social stratification (22) and thus affect the utilization of health care services. One study found that residence is a moderator between SES and health knowledge and that women with little education living in rural areas possess limited health knowledge (22). Another study found that race and sex were regulatory factors of the relationship between SES and cardiovascular disease risk (23). However, there seems to be some variation in these regulatory factors. In other studies, some common intermediate variables were not found to have an effect. For example, some researchers have found that the health status of older adults with low income or education levels is poor and that their age, insurance and comorbidities had no influence on this result (24). Few studies have examined the relationship between SES and PHC utilization and the possible intermediate variables involved.

We collected data in Jiangsu, a relatively economically developed province in eastern China. The GDP of Jiangsu Province in 2021 was 11,636.42 billion yuan, ranking second in China, and its per capita GDP reached 137,306 yuan, ranking third in China (25). The population of Jiangsu Province is also highly educated, as the province included 167 colleges or universities in 2021 (25). Jiangsu was also one of the first four pilot provinces to engage in comprehensive health care reform in China, and the development of PHIs in the province has been relatively effective (26). As early as 2013, 94.1% of individuals living in rural areas in Jiangsu Province were able to reach the nearest medical point within 15 min, and the “15-min health circle” in rural areas of the province was essentially achieved. In 2021, urban PHIs in Jiangsu Province provided diagnosis and treatment services for individuals, costing up to 69.91 billion (27).

Primary health care is widely regarded as the most equitable and cost-effective means to achieve universal health coverage (28). To encourage individuals to make greater use of PHC services, we conducted a survey on the utilization of PHI medical services in Jiangsu. The aims of this study were to analyze the relationship between individuals' SES and preference for PHIs and identify possible intermediary variables that affect residents' behavior.

## Methods

### Participants and sample size

A community-based, cross-sectional study was conducted in Jiangsu Province, China, from October 2021 to March 2022. Multistage sampling was used in this study. First, among the 13 cities in Jiangsu Province, we first selected the two cities with the highest (>2 trillion yuan) and lowest GDP (< 500 billion yuan) in Jiangsu Province, and then used a random number table to extract three cities from the remaining 11 cities. These five cities are concentrated in the southern region of Jiangsu Province. Second, three communities with different environmental conditions were selected by cluster random sampling from sample cities. We recruited by convenience sampling in the selected resident communities, and participants were those who lived continuously in the sample cities for more than 6 months, all of whom were over 18 years old.

We used the formula $\left(n={\left(Z_{\frac{\alpha }{2}}\right)}^{2}\times \frac{p\left(1-P\right)}{e^{2}}\right)$ for a single population to compute the sample size. The margin of error was set at 5% with a 95% confidence interval. According to the pilot survey, the proportion of people using PHC services was approximately 40%. The total sample size was calculated based on the assumption of a 40% level of choice. Therefore, the estimated sample size was 368. In addition, the effective rate of questionnaire completion was set at approximately 80%. Therefore, the minimum sample size for each city was 460, and the total minimum sample size was 3,680. A total of 5,000 questionnaires were distributed for the study, 4,234 of which were valid, resulting in an effective recovery rate of 84.68%. To study residents' preferences for medical services supplied by PHIs or hospitals, this study excluded subjects who medicated themselves (*n* = 2,817). The sample size was 1,417 (48.7% female; mean age: 44.41 ± 17.1 years).

### Data collection

The questionnaire was custom designed based on Andersen's behavioral model and expert consultation. After designing the questionnaire, we recruited 150 residents registered in Jiangsu Province to complete a pilot study to test the reliability and validity of the questionnaire and revised the questionnaire based on the survey results. After several revisions, the contents and structure of the questionnaire (Cronbach *a* = 0.74; KMO = 0.82) were determined. Data were collected via face-to-face interviews with trained interviewers, and respondents were given an incentive to motivate survey completion and thereby increase the response rate and validity of the data. In addition, to ensure the quality of the data, we adopt a logic proofreading and dual entry method during input. Before distributing the questionnaire, we described the study purpose and content to all respondents, obtained their informed consent to participate, and assured them that their privacy would be protected.

### Measures

#### Outcome variables

In this study, the outcome of interest was the answer to the following question: Are PHIs your first choice of medical services? There were two response options: “Yes” or “No” (yes = 1; no = 0). Because individuals who medicated themselves were excluded, “No” indicates that individuals choose hospitals instead of PHIs.

#### Independent variables

The explanatory variables were based on the conceptual framework of Andersen's behavioral model and categorized as follows:

- Predisposing: sociodemographic variables (age, gender, marital status, ethnicity) and individual preferences (part-time medical experts in PHIs, understanding of the first diagnosis from the PHIs rule, and opinion of PHIs).
- Enabling: education level, monthly income, occupation, current residential area, medical insurance, ability to reach a PHI within 15 min, and seen by a family doctor.
- Need: perceived health status, presence of chronic diseases, and perceived burden of medical payment.

### Individual-level SES

Since most articles were unclear regarding how measures of SES should be interpreted (29), we selected 17 Chinese and 29 English studies to investigate the factors affecting SES and selected the top 5 factors in order of frequency of mention. Five indicators (education, monthly income, current residential location, ethnicity, occupation) were entered into a principal component analysis (PCA), and a varimax (orthogonal) rotation was performed to generate individual-level SES. We extracted four components for calculating the comprehensive score of individual-level SES, and the cumulative variance explained was 90.67% (KMO = 0.619). Supplementary Table S1 shows the total variance in the sample explained by the first 4 components along with the eigenvalues assigned to each component. The four components were labeled comprehensive strength, development, earning ability, and living conditions. Individuals were divided into 3 classes (low, middle and high) based on synthetic principal component analysis.

### Intermediate determinants

According to the CSDH framework, material circumstances (including medical insurance, ability to reach a PHI within 15 min, and preference for part-time medical experts in PHIs), behaviors and biological factors (including the presence of chronic disease and seen by a family doctor), and psychosocial factors (including perceived health status, perceived burden of diseases, opinion of PHIs and understanding of the first diagnosis from the PHIs rule) were assumed to interact with individual-level SES.

### Statistical analysis

SPSS version 26.0 (IBM SPSS, Armonk NY, USA) and Stata version 17.0 (Stata Corp LP, College Station, TX, USA) were used for the statistical analysis. Descriptive statistics (frequencies and percentages) were calculated to describe the sociodemographic features of individuals (see Table 1). Ethnicity, education level, monthly income, occupation, and current residential location constitute SES, and they are inevitably related to SES. However, to demonstrate the distribution structure of each group on these 5 factors, these factors will be retained in Table 1. Then, univariate analysis with the chi-square (χ^2^) test was used to investigate the association between SES and the independent variables. Next, logistic regression analysis was conducted to examine relationships between the variables and results according to the model (see Table 2). The logistic regression was constructed based on the conceptual framework of Andersen's behavioral model by selecting specific variables. In the last step, an interaction analysis was performed to examine influences on the first choice of medical services. We used an α level ≤ 0.05 to indicate statistically significant differences in the above analysis.

**Table 1:** Characteristics and health behaviors of respondents by SES group (*n* = 1,417).

**Variable**	**Low SES**	**Moderate SES**	**High SES**	**χ^2^**	***P***
	***n* = 472**	***n* = 472**	***n* = 473**		
	**%**	**%**	**%**		
**Are PHIs your first choice of medical services?**					
Yes	58.3	46.8	33.4	58.87	< 0.01^**^
No	41.7	53.2	66.6		
**Gender**					
Male	47.7	50.0	56.2	7.42	< 0.05^*^
Female	52.3	50.0	43.8		
**Age group** ^a^					
18–44	45.6	52.8	60.5	69.34	< 0.01^**^
45–59	22.7	33.1	25.6		
≥60	31.8	14.2	14.0		
**Marital status**					
Unmarried	39.6	16.5	17.5	86.77	< 0.01^**^
Married	60.4	83.5	82.5		
**Ethnicity** ^b^					
Other	2.8	0.0	0.0	23.31	< 0.01^**^
Han Chinese	97.2	100.0	100.0		
**Preferred part-time medical experts in PHIs**					
Yes	67.6	66.3	71.2	2.87	0.24
No	32.4	33.7	28.8		
**Understanding of the first diagnosis from PHIs rule** ^c^					
Yes	35.6	41.5	50.7	22.51	< 0.01^**^
No	64.4	58.5	49.3		
**Opinion of PHIs**					
Poor	2.8	2.3	4.9	7.33	0.12
Neutral	35.8	35.2	31.1		
Good	61.4	62.5	64.1		
**Education level**					
Primary school or below	29.4	10.6	0.2	590.83	< 0.01^**^
Junior high school	26.5	27.3	8.7		
Senior high school	42.2	35.2	20.3		
Bachelor's degree	1.9	25.4	63.6		
Master's degree or above	0.0	1.5	7.2		
**Monthly income** ^b^					
< 1,000	47.7	3.8	0.2	770.64	< 0.01^**^
1,000-	5.9	5.9	0.2		
2,000-	33.1	32.6	17.1		
5,000-	12.5	47.2	51.6		
10,000-	0.8	10.2	30.4		
≥100,000	0.0	0.2	0.4		
**Occupation**					
Part-time employed or unemployed	35.0	9.3	0.0	986.31	< 0.01^**^
Laborer	54.0	29.2	0.2		
Self-employed	9.7	26.9	10.6		
Clerical staff	0.2	9.3	22.0		
Professional and technical staff	0.2	13.6	51.4		
Government administrator	0.8	11.7	15.9		
**Current residential location** ^d^					
Urban	26.7	57.0	98.7	518.92	< 0.01^**^
Rural	73.3	43.0	1.3		
**Medical insurance** ^e^					
URRMI	88.3	67.8	41.2	275.52	< 0.01^**^
UEBMI	8.1	30.1	58.1		
Other	3.6	2.1	0.6		
**Able to reach a PHI within 15 min**					
Yes	49.6	55.3	55.2	4.06	0.13
No	50.4	44.7	44.8		
**Seen by a family doctor**					
Yes	3.4	6.4	6.6	5.77	0.06
No	96.6	93.6	93.4		
**Perceived health status**					
Poor	24.8	16.7	18.2	11.04	< 0.05^*^
Normal	43.6	49.4	48.2		
Excellent	31.6	33.9	33.6		
**Have chronic diseases**					
Yes	43.2	42.4	39.3	1.63	0.44
No	56.8	57.6	60.7		
**Perceived burden of diseases**					
Slight	23.7	30.5	37.6	26.96	< 0.01^**^
Moderate	54.7	53.2	49.5		
Heavy	21.6	16.3	12.9		

^a^ The individuals were divided into three groups according to the standards of the World Health Organization as follows: young adult (18–44), middle-aged (45–59) and older adults (≥60). ^b^Ethnicity and monthly income were compared with a two-tailed Fisher's exact test. ^c^ China has implemented the rule that a first diagnosis should be made by a general practitioner in a PHI, but it has not yet become a necessary precondition for individuals to receive further treatment in hospitals. Not all individuals are aware of this rule. ^d^ We use the current residential location of respondents to determine whether they are rural residents or urban residents, not registered residence. ^e^ In mainland China, 95% of individuals are covered by two types of basic medical insurance, namely, urban and rural residents' medical insurance (URBMI) and urban employees' basic medical insurance (UEBMI). ^*^P < 0.05. ^**^P < 0.01.

**Table 2:** Logistic regression analysis results regarding the first choice of medical service.

**Variable**	**Are PHIs your first choice of medical services?**			
	**OR [95% CI]**	***P***	**AOR [95% CI]**	***P***
**SES**				
Low	Reference		Reference	
Moderate	0.63 [0.49–0.82]	< 0.01^**^	0.88 [0.64–1.19]	0.40
High	0.36 [0.28–0.47]	< 0.01^**^	0.69 [0.46–1.04]	0.08
**Gender**				
Male	Reference		Reference	
Female	1.07 [0.86–1.31]	0.56	1.06 [0.84–1.32]	0.64
**Age group**				
18–44	Reference		Reference	
45–59	1.16 [0.90–1.49]	0.25	1.03 [0.77–1.38]	0.84
>60	2.37 [1.79–3.14]	< 0.01^**^	1.79 [1.27–2.53]	< 0.01^**^
**Marital status**				
Unmarried	Reference		Reference	
Married	1.06[0.83–1.35]	0.65	0.90 [0.67–1.22]	0.49
**Preferred part-time medical experts in PHIs**				
Yes	Reference		Reference	
No	0.75 [0.60–0.94]	< 0.05^*^	0.70 [0.55–0.90]	< 0.01^**^
**Understanding of the first diagnosis from PHIs rule**				
Yes	Reference		Reference	
No	1.16 [0.94–1.43]	0.17	1.04 [0.82–1.31]	0.75
**Opinion of PHIs**				
Poor	Reference		Reference	
Neutral	1.29 [0.70–2.38]	0.42	1.26 [0.66–2.43]	0.49
Good	1.46 [0.80–2.67]	0.22	1.44 [0.76–2.74]	0.27
**Current residential location**				
Rural	Reference		Reference	
Urban	0.32 [0.26–0.40]	< 0.01^**^	0.41 [0.30–0.56]	< 0.01^**^
**Medical insurance** ^a^				
URBMI	Reference		Reference	
UEBMI	0.81 [0.64–1.01]	0.06	1.14 [0.87–1.49]	0.36
Other	1.91 [0.90–4.05]	0.09	2.44 [1.11–5.33]	< 0.05^*^
**Able to reach a PHI within 15 min**				
Yes	Reference		Reference	
No	1.17 [0.95–1.45]	0.14	1.09 [0.87–1.36]	0.47
**Seen by a family doctor**				
Yes	Reference		Reference	
No	1.69 [1.06–2.69]	< 0.05^*^	1.59 [0.96–2.61]	0.07
**Perceived health status**				
Poor	Reference		Reference	
Normal	1.24 [0.94–1.64]	0.13	1.25 [0.92–1.69]	0.16
Excellent	1.09 [0.81–1.47]	0.56	1.07 [0.76–1.50]	0.70
**Have chronic diseases**				
Yes	Reference		Reference	
No	0.79 [0.64–0.98]	< 0.05^*^	1.036 [0.79–1.36]	0.79
**Perceived burden of diseases**				
Slight	Reference		Reference	
Moderate	1.08[0.85–1.37]	0.51	0.98 [0.76–1.27]	0.89
Heavy	1.17[0.85–1.60]	0.34	0.91 [0.64–1.30]	0.61

^a^ The independent variable was removed in the stepwise regression of Model 2. ^*^P < 0.05. ^**^P < 0.01.

## Results

Table 1 presents the distribution of various factors among the SES groups. Only 33.4% of the high-SES group reported that PHIs were their first choice for medical services, but the proportions in the other two groups were larger (*p* < 0.01). The high-SES group included a slightly larger proportion of men (*p* < 0.05) and was younger than the other two groups (*p* < 0.01). Over 80% of respondents in the moderate- and high-SES groups were married, which is a higher proportion than in the low-SES group (*p* < 0.01). Only 2.8% of the low-income group was from another ethnic group (*p* < 0.01). More respondents in the high-SES group preferred to receive treatment from part-time medical experts in PHIs (*p* > 0.05) and understood the first diagnosis from the PHIs rule (*p* < 0.01) compared to the other two groups. More than 70% of individuals in the high-SES group had a bachelor's degree or above, and 80% of them earned over 5,000 yuan monthly (*p* < 0.01). More respondents in the low- and moderate-SES groups lived in rural areas (*p* < 0.01). The proportions of respondents with urban and rural residents' medical insurance (URRMI) were as follows: low- (88.3%), moderate- (67.8%), and high-SES groups (41.2%). This difference among groups was statistically significant (*p* < 0.01). Approximately half of the respondents in the three groups could reach a PHI after a 15-min walk (*p* > 0.05). The proportions of respondents seen by family doctors in the three groups were 3.4, 6.4, and 6.6, respectively, which are low (*p* > 0.05). More respondents in the low-SES group had poor perceived health (*p* < 0.05). Approximately 40% of respondents had chronic diseases (*p* > 0.05). More respondents in the low-SES group perceived the burden of disease to be heavy (*p* < 0.01).

Table 2 shows the results of logistic regression models examining independent variables associated with the first choice of medical services. In univariate logistic regression analysis, factors associated with outcome variables included SES, age, preference for part-time medical experts in PHIs, current residential location, being seen by a family doctor, and having chronic diseases. However, after controlling for other variables, the association between SES and choice of PHIs was not statistically significant (*p* > 0.05). Older adults and respondents who were not covered by basic medical insurance preferred to seek medical services from PHIs (*p* < 0.01). Those who had no preference for part-time medical experts in PHIs and urban individuals sought hospitals as their first choice of medical service (*p* < 0.01). In addition, we found that no associations of the first choice of medical services with other factors were significant, which included gender, marital status, understanding of the first diagnosis from the PHIs rule, opinion of PHIs, ability to reach a PHI within a 15-min walk, seen by a family doctor, perceived health status, the presence of chronic diseases, and perceived burden of disease (*p* > 0.05).

According to the CSDH framework, we tested the interactions of SES with material circumstances, behaviors and biological factors, and psychosocial factors. As shown in Table 3, individual-level SES interacted with all three types of factors mentioned above. Respondents in the moderate-SES group covered by UEBMI and those in the high-SES group covered by basic medical security tend not to prefer PHIs. Residents who could not reach PHIs within 15 min were more likely to prefer PHIs as their first choice for medical services, while the high-income group was the opposite. Respondents with low income and no preference for part-time experts, as well as those in moderate- and high-SES groups, were less likely to prefer medical services provided by PHIs. Compared to low-SES individuals with chronic diseases, those without chronic diseases were less likely to choose PHIs as the first option (*p* < 0.01). Surprisingly, respondents in the middle- and high-SES groups who were seen by family doctors were also more likely not to choose the medical services provided by PHIs (*p* < 0.01). Individuals in the moderate-SES group with poor health and those in the high-SES group were more likely not to choose PHIs (*p* < 0.01). Respondents in the high-SES group, regardless of whether they had a heavy disease burden, tended not to choose the medical services provided by PHIs (*p* < 0.01). High-income individuals with poor health tended not to choose PHIs (*p* < 0.05).

**Table 3:** Multiplicative interaction analysis results regarding the first choice of medical services.

**Variable**		**OR [95% CI]**	***P***
**Material circumstances**			
SES × Medical insurance			
SES	Medical insurance		
Low	URBMI	1	
	UEBMI	1.00 [0.51–1.97]	0.99
	Other	1.75 [0.61–5.07]	0.30
Moderate	URBMI	0.55 [0.41–0.74]	< 0.01^**^
	UEBMI	0.87 [0.59–1.27]	0.46
	Other	1.10 [0.30–3.94]	0.89
High	URBMI	0.36 [0.25–0.51]	< 0.01^**^
	UEBMI	0.37 [0.27–0.51]	< 0.01^**^
	Other	0.37 [0.03–4.05]	0.41
SES × Able to reach a PHI within 15 min			
SES	Able to reach a PHI within 15 min		
Low	Yes	1	
	No	1.54 [1.07–2.22]	< 0.05^*^
Moderate	Yes	0.71 [0.50–1.01]	0.06
	No	0.88 [0.61–1.28]	0.50
High	Yes	0.51 [0.35–0.73]	< 0.01^**^
	No	0.38 [0.25–0.55]	< 0.01^**^
SES × Preferred part-time medical experts in PHIs			
SES	Preferred part-time medical experts in PHIs		
Low	Yes	1	
	No	0.62 [0.42–0.91]	< 0.05^*^
Moderate	Yes	0.56 [0.41–0.77]	< 0.01^**^
	No	0.49 [0.34–0.73]	< 0.01^**^
High	Yes	0.34 [0.25–0.47]	< 0.01^**^
	No	0.23 [0.15–0.35]	< 0.01^**^
**Behaviors and biological factors**			
SES × Have chronic diseases			
SES	Have chronic diseases		
Low	Yes	1	
	No	0.46 [0.32–0.68]	< 0.01^**^
Moderate	Yes	0.41 [0.27–0.61]	< 0.01^**^
	No	0.40 [0.27–0.59]	< 0.01^**^
High	Yes	0.21 [0.13–0.32]	< 0.01^**^
	No	0.24 [0.17–0.36]	< 0.01^**^
SES × Seen by a family doctor			
SES	Seen by a family doctor		
Low	Yes	1	
	No	1.20 [0.43–3.36]	0.73
Moderate	Yes	0.61 [0.47–0.80]	< 0.01^**^
	No	1.08 [0.51–2.30]	0.84
High	Yes	0.34 [0.26–0.44]	< 0.01^**^
	No	0.88 [0.42–1.82]	0.72
**Psychosocial factors**			
SES × Perceived health status			
SES	Perceived health status		
Low	Poor	1	
	Normal	1.20 [0.76–1.90]	0.43
	Excellent	1.23 [0.75–2.00]	0.41
Moderate	Poor	0.45 [0.25–0.82]	< 0.01^**^
	Normal	0.88 [0.56–1.37]	0.57
	Excellent	0.69 [0.43–1.12]	0.14
High	Poor	0.42 [0.24–0.75]	< 0.01^**^
	Normal	0.45 [0.28–0.71]	< 0.01^**^
	Excellent	0.37 [0.22–0.61]	< 0.01^**^
SES × Perceived burden of diseases			
SES	Perceived burden of diseases		
Low	Slight	1	
	Moderate	1.52 [0.97–2.38]	0.07
	Heavy	1.38 [0.80–2.37]	0.25
Moderate	Slight	0.99 [0.61–1.63]	0.98
	Moderate	0.72 [0.46–1.12]	0.15
	Heavy	1.09 [0.61–1.96]	0.75
High	Slight	0.53 [0.33–0.86]	< 0.01^**^
	Moderate	0.50 [0.32–0.79]	< 0.01^**^
	Heavy	0.31 [0.16–0.63]	< 0.01^**^
SES × Opinion of PHIs			
SES	Opinion of PHIs		
Low	Poor	1	
	Neutral	1.02 [0.33–3.18]	0.97
	Good	1.32 [0.43–4.03]	0.62
Moderate	Poor	2.29 [0.41–12.73]	0.35
	Neutral	0.67 [0.22–2.09]	0.49
	Good	0.77 [0.25–2.36]	0.65
High	Poor	0.13 [0.03–0.66]	< 0.05^*^
	Neutral	0.43 [0.14–1.34]	0.15
	Good	0.46 [0.15–1.41]	0.17
SES × Understanding of the first diagnosis from PHIs rule			
SES	Understanding of the first diagnosis from PHIs rule		
Low	Yes	1	
	No	1.35 [0.92–1.97]	0.13
Moderate	Yes	0.71 [0.47–1.07]	0.10
	No	0.81 [0.55–1.18]	0.27
High	Yes	0.50 [0.34–0.75]	< 0.01^**^
	No	0.37 [0.25–0.56]	< 0.01^**^

^*^P < 0.05. ^**^P < 0.01.

## Discussion

Certain segments of the population, such as older adults, patients with chronic diseases, individuals living in rural areas, and other vulnerable groups, may face health inequities (30–32). PHC is an effective way of compensating for health equity problems and a cornerstone of a sustainable health system that provides universal health coverage (33). In this study, older adults, rural residents, individuals lacking coverage by basic medical insurance and those with a preference for part-time medical experts in PHIs in Jiangsu Province were more likely to choose medical services supplied by PHIs. PHIs mainly attract these four groups of individuals due to their accessibility, use of simple procedures for medical services and low prices, which are important components of health service accessibility (34, 35). The proportion of older adults with chronic diseases was found to exceed 75% (36), while rural individuals have experienced the migration of the labor force to cities; the proportion of older adults in rural areas is also significantly higher than that in urban areas (37). These individuals typically have lower levels of SES (38). According to the sixth national health service statistical survey report, the coverage rate of basic medical security in China has exceeded 95% (39). The unemployed, migrant workers, and freelancers are the main constituent individuals who do not participate in medical insurance. They also have lower SES. In addition, in China, high-quality medical resources are limited. The probability of making appointments with some medical experts at hospitals is low, or keep on waiting for the appointment. Therefore, accessing those medical experts through PHIs is a very convenient way for residents with expert preferences. Individuals from these groups may target visitors to PHIs, and meeting their needs should be the focus of the development and construction of PHIs.

Material circumstances such as medical insurance, ability to reach a PHI within a 15-min walk, and preference for part-time medical experts in PHIs were all related to China's medical policies, which impact individuals' choices. Although China's basic medical insurance covers over 95% of residents (40), there are still a small number of individuals who remain without coverage for various reasons. Therefore, the affordable and easily accessible medical services supplied by PHIs motivate individuals to select them as their first choice. Based on the interaction between medical insurance and SES, those with better economic conditions tended not to choose PHI medical services. From the data, it seems that this choice is due to economic pressure. Similar selection patterns were also revealed in the analysis of the ability to reach a PHI within a 15-min walk × SES interaction and the preference for part-time medical experts in PHIs × SES interaction. It is worth considering why individuals choose to seek medical services at hospitals when they have good economic conditions. Previous studies have shown that the perceived low competency of medical personnel, outdated medical facilities, and low-quality services have caused many individuals to bypass PHC (41, 42). An earlier study also showed that PHC plays an important role by meeting the needs of individuals (43). Improving service quality and focusing on family health care needs will help increase the utilization of PHC services as well as the effectiveness and efficiency of the health system (8).

Leadership from family physicians can enable the delivery of high-quality primary health care that is accessible, comprehensive, coordinated, continuous and person-centred (44). However, in this study, among the 1,417 respondents, only 77 had been seen by a family doctor. This is because the proportion of respondents who signed contracts with family doctors is lower, and the utilization rate of health services supplied by family doctors is also lower. Surprisingly, being seen by a family doctor did not increase respondents' tendency to choose PHI medical services. A Chinese study showed that being seen by a family doctor improved patients' perceptions of PHC quality in pilot cities (45). Our study showed that individuals who viewed PHIs as satisfactory did not increase their willingness to choose PHIs. At present, there is no strict requirement in China that patients must be referred by doctors in PHIs before they visit a doctor in the hospital. This means that patients can directly go to the hospital for treatment as needed. In China, residents had long been accustomed to going to hospitals for medical treatment. Therefore, more attractive measures are needed to transform the medical behaviors of residents. In fact, only 20% of patients who obtained a first diagnosis from a PHI in Jiangsu were found to have signed a contact with a family doctor (46). Therefore, it is still necessary to investigate ways to promote PHI services in Jiangsu in terms of family doctor use.

SES did not have a significant effect on the utilization of PHI medical services. However, SES was found to interact with variables in the CSDH framework to influence the probability of choosing PHI medical services. For the same factor, individuals with different SES showed different patterns of PHI medical service use. Three groups of factors (material circumstances, behaviors and biological and psychosocial factors) interacted with SES to influence selections. The choices of the high-SES group were consistent: high-income individuals were less likely to choose medical services provided by PHIs. Many studies have confirmed that SES has a significant impact on people's physical health (47–50). A person's ability to access health care is mediated by SES (51). Superior social and economic conditions provide individuals with high SES with more choices, especially because China's medical system does not limit patients' access to health resources at different levels. Therefore, it is necessary to be aware that PHIs only serve impoverished individuals and are not completely able to reduce medical costs.

Compared to other people, low-income individuals with chronic diseases were more willing to choose PHIs. Chronic diseases require long-term treatment, which takes a long time and is expensive (52). In low- and middle-income countries, the monthly treatment cost for hypertension is approximately $22, while the costs for stroke and coronary heart disease are much higher, at approximately $300 to $1,000 per month (53). In China, the medical service costs of primary health institutions are lower, and the reimbursement ratio is higher. An earlier study found that a greater average reimbursement rate in PHIs was associated with a 73% lower probability of visiting municipal- and higher-level hospitals (54). Therefore, SES will have a greater impact on patient selection. In addition, it is interesting that high-income individuals, regardless of their health status, disease burden, and understanding of the first diagnosis rule, tended not to choose PHI medical services. This confirms the above discussion. From this, it can be seen that the medical services provided by PHIs have not yet received widespread recognition (7, 55).

## Limitation

Several factors may have limited our study. As we conducted a cross-sectional study, our conclusions only pertain to the correlations between factors, and we cannot make causal inferences. Our sampling method was not completely random at the community level, which may have resulted in inestimable bias. Finally, as some questions included in the survey focused on residents' health behaviors over the past year, the data may have been affected by memory bias.

## Conclusions

Individuals who are older, live in rural areas, and have chronic diseases are the target visitors for PHIs. SES, as a single factor, did not impact whether PHI medical services were chosen, but it mediated the relationships with other factors. When developing PHI services, it is necessary to pay attention to the needs of key populations. After controlling for other factors, individuals with high SES may make different choices from those with low or moderate SES in terms of PHI medical service utilization.

## Data availability statement

The raw data supporting the conclusions of this article will be made available by the authors, without undue reservation.

## Ethics statement

The studies involving human participants were reviewed and approved by Xuzhou Medical University of Ethics Committee. The studies were conducted in accordance with the local legislation and institutional requirements. Written informed consent to participate in this study was provided by the participants.

## Author contributions

CM: Investigation, Funding acquisition, Writing – original draft, Writing – review & editing. XF: Writing – original draft, Writing – review & editing. HS: Writing – original draft, Writing – review & editing. YY: Investigation, Writing – original draft, Writing – review & editing. BL: Writing – original draft, Writing – review & editing. WS: Writing – original draft, Writing – review & editing. JC: Methodology, Writing – original draft, Writing – review & editing. XH: Conceptualization, Funding acquisition, Writing – original draft, Writing – review & editing.
