# Supplementary material for: The relationship between individual-level socioeconomic status and preference for medical service in primary health institutions: a cross-sectional study in Jiangsu, China
Source: Front Public Health. 2024 Jan 11;11:1302523. doi: 10.3389/fpubh.2023.1302523 (PMC10809986; doi:10.3389/fpubh.2023.1302523)
Supplement: Supplementary file 1 [file Table_1.DOCX]

**Supplementary table S1: The results of principal component analysis**

| Component | Eigenvalue | Different | Proportion | Cumulative |
| --- | --- | --- | --- | --- |
| 1 | 1.958 | 0.934 | 0.392 | 0.392 |
| 2 | 1.024 | 0.565 | 0.205 | 0.597 |
| 3 | 0.968 | 0.385 | 0.194 | 0.790 |
| 4 | 0.583 | 0.116 | 0.112 | 0.907 |
| 5 | 0.467 |  | 0.093 | 1.000 |
